# Antibiotic Resistance Hotspot: Comparative Genomics Reveals Multiple Strains of Multidrug-Resistant *Citrobacter portucalensis* in Edible Snails

**DOI:** 10.3390/ijms25189889

**Published:** 2024-09-13

**Authors:** Arthur C. Okafor, Adriana Cabal Rosel, Frank C. Ogbo, Charles O. Adetunji, Odoligie Imarhiagbe, Lukas Gamp, Anna Stöger, Franz Allerberger, Werner Ruppitsch

**Affiliations:** 1 Institute of Medical Microbiology and Hygiene, Austrian Agency for Health and Food Safety, 1090 Vienna, Austria; 2 Department of Applied Microbiology and Brewing, Nnamdi Azikiwe University, Awka PMB 5025, Anambra State, Nigeria; 3 Department of Microbiology, Edo State University, Uzairue PMB 04, Edo State, Nigeria; 4 Department of Health and Social Science, London School of Science and Technology, Birmingham B6 5RQ, UK; 5 Department of Biotechnology, University of Natural Resources and Life Sciences, 1180 Vienna, Austria; 6 Faculty of Food Technology, Food Safety and Ecology, University of Donja Gorica, 81000 Podgorica, Montenegro

**Keywords:** snails, *Citrobacter portucalensis*, genome sequencing, cgMLST, antimicrobial resistance genes

## Abstract

The demand for terrestrial snails as a food source is still on the increase globally, yet this has been overlooked in disease epidemiology and the spread of antimicrobial resistance. This study conducted genomic analyses of twenty *Citrobacter portucalensis* strains isolated from live edible snails traded in two hubs. The isolates were subjected to MALDI-TOF MS, antimicrobial resistance testing, whole genome sequencing, and analyses for in-depth characterization. The findings disclosed that seventeen strains across the two trading hubs were distinct from previously reported ones. Four isolates were found to share the same sequence type (ST881). Genome-based comparison suggests a clonal transmission of strains between snails traded in these hubs. All the isolates across the two hubs harbored similar variety of antimicrobial resistance genes, with notable ones being *bla*CMY and *qnrB*. Sixteen isolates (80%) expressed phenotypic resistance to second-generation cephalosporins, while eleven isolates (55%) exhibited resistance to third-generation cephalosporins. This report of multi-drug-resistant *C. portucalensis* strains in edible snails highlights significant concerns for food safety and clinical health because of the potential transmission to humans. Enhanced surveillance and stringent monitoring by health authorities are essential to evaluate the impact of these strains on the burden of antimicrobial resistance and to address the associated risk.

## 1. Introduction

Some terrestrial snails are considered nutritious and are commonly available in the market for commercial purposes in most regions of the world. *Achatina achatina* is a terrestrial gastropod of the family “Achatinidae”, which has been listed among edible snails [1]. The demand for terrestrial snails as a food source is still on the increase among consumers. For instance, the European market size for such snails is estimated to grow to USD 699.72 million by 2028 [2]. The association of edible snails with high counts of viable bacterial pathogens, which can be difficult to eliminate during culinary preparation in domestic kitchens [3,4,5], is a neglected factor in disease epidemiology and the spread of antimicrobial resistance. This represents a serious health concern in countries where the value chain is neither traceable nor controlled.

*Citrobacter* species are members of the Enterobacteriaceae, which are of rising clinical importance and usually present in soil, water, and the intestines of food animals, such as snails [3,6,7,8]. Pawar et al. concluded that an apparent feature of bacterial communities in snails’ gastrointestinal tract was the abundance of members of the genus *Citrobacter* [9]. There is no report on the genomic diversity of specific strains of *Citrobacter* in terrestrial snails destined for human consumption which might aid in assessing the safety of the value chain. The use of conventional tests and MALDI-TOF MS for differentiating species of *Citrobacter* for recognizing species of clinical significance has been challenging [8,10,11], thereby highlighting the need for more-advanced techniques. The whole genome sequence (WGS)-based characterization of bacterial pathogens offers unique resolution in discriminating even highly related lineages, thereby precluding the use of species-dependent protocols. Genome data provide more information concerning pathogen detection and identification, epidemiological typing, and antimicrobial resistances [12]. Several *Citrobacter* species have been found to be zoonotic pathogens capable of transmission to humans during the processing of carcasses of food animals; they can survive long periods of time in their hosts and accumulate antimicrobial resistance (AMR) genes [13,14].

*C. portucalensis* was first isolated from an aquatic sample in Portugal [15]. WGS had been used to detect *C. portucalensis* in non-clinical sources such as water and vegetables and has been found to possess antimicrobial resistance [15,16]. A pioneer report on a clinical multi-drug-resistant *C. portucalensis* strain suggests that the prevalence of this species in the clinical environment is significantly underrated. This study emphasized the potential for clinically “rare” species to become reservoirs of drug resistance in the future [17]. To our knowledge, WGS has not been deployed to assess the genomic diversity of multiple strains of *C. portucalensis* associated with non-clinical sources in the context of food safety.

Hence, our study aimed to understand the diversity and genomic characteristics of twenty *C. portucalensis* strains recovered from the gut of live terrestrial snails fated for culinary preparations from two popular trading hubs across two cities in Nigeria. Our study contributes specific data for enhancing public health surveillance, thereby engendering efforts to reduce the burden of AMR as per the One Health concept.

## 2. Results and Discussion

Our study has demonstrated the emergence of diverse strains of *C. portucalensis* in the gut of terrestrial snails sold as food source in two markets. The twenty *C. portucalensis* isolates were assigned to seventeen new sequence types (STs) (ST881–ST897) based on the conventional MLST scheme. Four isolates (Nsk1, Nsk2, Igbk10, and Igbk13) were found to share the same ST (ST881) (Table 1). This diversity across the two markets supports our hypothesis that occurrence and spread of *C. portucalensis* in food snails is underestimated with conventional methods. Previous studies have reported the presence of *C. portucalensis* in non-clinical sources such as water and vegetables via WGS analysis [15,16]. *C. portucalensis* was detected in some Nigerian vegetables [16]. Vegetables play a major role in the dietary habit of snails [18] and could be the source of this bacterium in snails.

**Figure 1:**
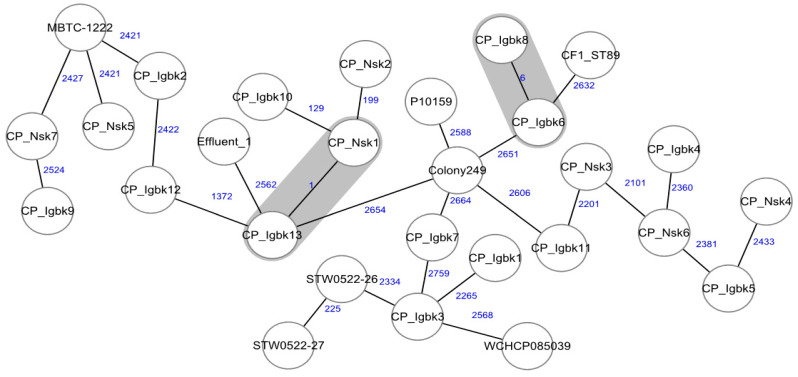
Minimum spanning tree generated from the cgMLST comparison of *Citrobacter portucalensis* strains associated with edible snails (n = 20) with other previously reported strains (n = 8) available on GenBank. The numbers close to the lines indicate allelic difference.

The genetic relationship between the isolates in our study, determined using conventional MLST and an ad hoc cgMLST scheme, resulted in a cluster between the markets for isolates Nsk1 and Igbk13 (Figure 1 and Table 1). The allelic difference between the two isolates is 1 (Figure 1), which suggests a clonal transmission of strains between snails traded in two markets. Genomic comparisons utilizing cgMLST can be effective for detecting novel transmission [19]. All the strains in our study were found to be unrelated to the GenBank strains.

All strains harbored more than 20 AMR genes, notably *bla*CMY and *qnrB* (Table 2). This is a first-time report of the presence of AMR genes in *C. portucalensis* strains associated with terrestrial snails meant for culinary processing in domestic kitchens. These findings are of clinical significance because a previous study had reported a similar resistant strain of *C. portucalensis* (strain 3839) recovered from a diabetic patient in China [17]. *bla*CMY and *qnrB* encode resistance to beta-lactam and fluoroquinolone antibiotics, respectively. These genes (*bla*CMY and *qnrB*) have also been reported in *C. portucalensis* strains isolated from vegetables, poultry, diabetic patients, and green sea turtles [16,17,20,21]. Thus, our results corroborate the findings of other studies that *C. portucalensis* is a global multi-drug-resistant bacterium that harbors intrinsic resistance genes, such as *bla*CMY and *qnrB* [21], with potential for mobilization to mobile genetic elements [15,22]. Scientific investigations have recognized soil as a principal reservoir for antibiotic resistance genes [23,24,25]. The soil is probably the source of the AMR genes harbored by these snails because their movement is characterized by continuous contact with the soil. Our study also demonstrated that the isolates exhibited phenotypic resistance to certain antibiotics, such as cefoxitin (n = 16), ampicillin (n = 9), amoxicillin/clavulanic acid (n = 12), and cefpodoxime (n = 11), which have food safety and clinical implications. One of our strains (Igbk10) was found to be deceptive because of its inability to express AMR phenotypically despite its possession of functional genes and plasmids (Table 2).

Three virulence genes (*traT*, *irp2*, and *fyuA*) were concurrently detected in the genomes of 4 of the 20 isolates (Table 2) of the same sequence type (ST881) (Table 1). The *traT* increases serum resistance in the regulation of virulence in bacterial pathogens [26,27], while *irp2* and *fyuA* are iron-uptake genes [28]. Salgueiro et al. [29] reported some virulence genes, such as *traT*-type, present in strains of *C. portucalensis* isolated from bivalve samples collected in Portuguese farms. *traT* was the most-detected virulence gene (9.0%) in 66 Gram-negative bacteria isolated from seabream and bivalve molluscs in the southern region of Portugal. *fyuA* and *irp2* were among the nine virulence genes demonstrated to occur more frequently in avian pathogenic *E. coli* strains than non-pathogenic strains [30]. In fact, these two genes have been detected by PCR in *E. coli* strains isolated from poultry birds with clinical signs of colibacillosis in Korea and Brazil [31,32]. Exploring the distribution of virulence genes among *C*. *portucalensis* isolates can offer valuable insights into their epidemiology and pathogenic potential.

Two plasmids (col440I and IncFIB(k)) were detected in one of our 20 isolates. Plasmid IncFIB(pHCM2) was found in another isolate (Table 2). IncFIB(K)-type plasmid has been reported in strains of *C. portucalensis* recovered from gills of fishes in the southern region of Portugal [29]. IncFIB(K)-type and Col440I-type plasmids have been described for strains of *C. portucalensis* recovered from bivalve samples collected in farms in the southern region of Portugal [29]. The detection of different plasmid types among these *C. portucalenesis* strains underscores their genetic diversity and adaptability.

## 3. Materials and Methods

### 3.1. Isolation of Citrobacter

A total of 50 samples of terrestrial snails (*Achatina achatina*) were randomly procured from two markets (Nsukka and Igboukwu) in the southeast zone of Nigeria. The intestinal sections of the samples were aseptically collected during the usual evisceration step of culinary preparation in the domestic kitchen, as is common practice in Nigeria. Within an hour, samples were transported to the laboratory for analysis. The intestinal samples (50 g) were homogenized with 450 mL of peptone water (Oxoid, UK). Aliquots (5 mL) of the homogenate were enriched in Selenite F broth (45 mL) for 18 h before plating 0.1 mL on Salmonella–Shigella agar (Oxoid). Strains were identified as *Citrobacter* using standard bacteriological methods, as previously described [3].

### 3.2. In Vitro Characterization of Sensitivity to Antimicrobial Agents

The isolates were tested for antimicrobial resistance using the disk diffusion method according to the CLSI M02-A12 Supplement [33]. The following antimicrobial agents (Oxoid) were used: ampicillin (10 µg), imipenem (10 µg), meropenem (10 µg), mecillinam (10 µg), piperacillin/tazobactam (110 µg), amoxycillin/clavulanic acid (30 µg), gentamicin (10 µg), ertapenem (10 µg), levofloxacin (5 µg), ciprofloxacin (5 µg), trimethoprim (5 µg), amikacin (30 µg), cefpodoxime (10 µg), cefpodoxime/clavulanic acid (1 µg), cefoxitin (30 µg), cefotaxime (5 µg), cefepime (30 µg), and ceftazidime (30 µg). The European Committee on Antimicrobial Susceptibility Testing [34] Clinical Breakpoint Table was used for interpretation.

### 3.3. Identification of Bacteria Using MALDI-TOF MS

Twenty strains that were found to be either hemolytic or resistant to at least one antibiotic were selected for identification using matrix-assisted laser desorption ionization–time-of-flight mass spectrometry (MALDI-TOF MS) based on the manufacturer’s instructions (Bruker Daltonics, Bremen, Germany). Briefly, isolates were sub-cultured on blood agar and incubated at 35 °C for 24 h prior to the MALDI-TOF identification step. Next, a single colony of each isolate was picked from the agar plate and aseptically smeared on a spot of a bar-coded MSP 96 target polished steel plate (Bruker Daltonics, Germany). The smeared spots on the bar-coded steel plate were overlaid with 1 µL matrix solution provided by the manufacturer and allowed to dry at room temperature. The samples were measured with a Bruker Microflex MALDI-TOF Mass Spectrometer (Bruker Daltonics, Germany) using MBT AutoX. Identification was conducted using the Biotyper software, version 4.1.100, in the default settings. Identification scores of >1.7 or >2.0 indicated a reliable genus or species identification, respectively [35].

### 3.4. Whole Genome Sequencing (WGS) of the Isolates and Analysis

Genomic DNA was extracted from overnight cultures grown at 37 °C on blood agar using the MagAttract^®^ HMW DNA kit 48 (Qiagen, Hilden, Germany) according to the manufacturer’s instructions. The concentration of DNA was measured using Qubit 3.0 fluorometer (Thermo Fisher Scientific, Waltham, MA, USA) with the dsDNA HS assay kit (Thermo Fisher Scientific) and DropSense 16 System (Trinean NV, Gentbrugge, Belgium) in accordance with the manufacturer’s manual.

In strict compliance with the manufacturer’s protocol, ready-to-sequence DNA libraries were prepared using the Nextera XT DNA library preparation kit (Illumina, San Diego, USA). WGS (paired end, 2 × 150 bp) was performed using the Illumina Nextseq 2000 instrument. The de novo assembly of raw reads was conducted with SPAdes (version 3.9.0) [36]. The WGS data were analyzed using multi-loci sequence typing (MLST) and core genome (cg)MLST using Ridom SeqSphere^+^ software v.9.0.8 (Ridom GmbH, Münster, Germany). An ad hoc cgMLST scheme comprising 2962 core target genes was created using *C. portucalensis* NCTC11104 as the reference genome (GenBank accession NZ_LR134214.1), along with 21 other *C. portucalensis* genomes from GenBank, as query genomes for comparison of our strains with all *C. portucalensis* genomes available at NCBI GenBank. The genomic relationship of isolates was depicted using minimum spanning trees.

The presence of resistance genes, virulence genes, and mobile genetic elements were determined with the Comprehensive Antibiotic Resistance Database (CARD) (https://card.mcmaster.ca) (accessed on 14 July 2022) and Centre for Genomic Epidemiology web server tools (https://cge.food.dtu.dk/services/MobileElementFinder/) (accessed on 19 July 2022). All software was used with default parameters.

## 4. Conclusions

The presence of multi-drug-resistant strains of *C. portucalenesis* in edible snails traded in two Nigerian hubs, as determined by next-generation sequencing, is of both food safety and clinical concern for areas where the value chain of snails is neither traceable nor controlled. These findings highlight the need for enhanced surveillance and stringent monitoring of edible snails to mitigate potential risks associated with the spread of antimicrobial resistance. Public health authorities should prioritize assessing and managing the impact of these resistant strains to prevent their potential transmission to humans and address the broader implications for antimicrobial resistance.

## Figures and Tables

**Table 1:** Selected genomic features of *Citrobacter portucalensis* isolates (n = 20) from snails sold in two Nigerian markets.

Isolate	Source Location	Size (bp)	GC Content (%)	Sequence Type	Average Coverage	% Good Targets cgMLST	Genome Accession Number
CP_Nsk1	Nsukka	5,252,065	51.8	881	41	99.2	JALGBJ000000000
CP_Nsk2	Nsukka	5,170,058	52.0	881	46	99.0	JANBWY000000000
CP_Nsk3	Nsukka	4,896,645	52.0	882	53	99.2	JANBWX000000000
CP_Nsk4	Nsukka	4,969,172	52.0	883	69	98.9	JANBWW000000000
CP_Nsk5	Nsukka	5,136,318	52.0	895	67	99.4	JANBWN000000000
CP_Nsk6	Nsukka	5,012,648	51.9	896	54	99.2	JANBWM000000000
CP_Nsk7	Nsukka	4,843,198	52.0	897	53	99.3	JANBWL000000000
CP_Igbk1	Igboukwu	4,648,030	52.0	884	82	99.6	JANBWV000000000
CP_Igbk2	Igboukwu	4,968,643	51.8	885	92	99.3	JANBWU000000000
CP_Igbk3	Igboukwu	4,766,390	52.0	886	68	98.9	JANBWT000000000
CP_Igbk4	Igboukwu	4,838,025	52.1	887	75	99.5	JANDBI000000000
CP_Igbk5	Igboukwu	4,943,623	52.1	888	67	98.9	JANDBH000000000
CP_Igbk6	Igboukwu	5,797,490	51.8	889	73	99.0	JANBWS000000000
CP_Igbk7	Igboukwu	5,295,614	51.7	890	47	98.1	JANDBG000000000
CP_Igbk8	Igboukwu	4,794,523	52.1	891	83	99.3	JANBWR000000000
CP_Igbk9	Igboukwu	5,430,528	51.5	892	61	98.6	JANBWQ000000000
CP_Igbk10	Igboukwu	5,192,886	51.9	881	82	98.8	JANBWP000000000
CP_Igbk11	Igboukwu	4,937,101	52.0	893	49	99.3	JANBWO000000000
CP_Igbk12	Igboukwu	4,960,921	51.8	894	77	99.2	JANDBF000000000
CP_Igbk13	Igboukwu	5,162,285	51.9	881	38	98.6	JANBWK000000000

cgMLST: core genome multi-locus sequence typing.

**Table 2:** Antimicrobial resistance, virulence, and plasmids detected in *Citrobacter portucalensis* isolates (n = 20) from snails sold in two Nigerian markets.

Isolate	Resistance Phenotype	Resistance Genes	Virulence Genes	Plasmid
CP_Nsk1	AMC-CPD-FOX	msbA, kdpE, PmrF, baeR, mdtC, mdtB, mdfA, marA, rsmA, KpnE, KpnF, CRP, ampH, emrB, emrR, H-NS, mdtG, UhpT, PBP3, GlpT, EF-Tu, soxS, marR, acrA, QnrB6, CMY-46.	traT, irp2, fyuA	-
CP_Nsk2	CN-CPD-FOX	msbA, kdpE, PmrF, baeR, mdtC, mdtB, mdfA, marA, rsmA, KpnE, KpnF, CRP, ampH, emrB, emrR, H-NS, mdtG, UhpT, PBP3, GlpT, EF-Tu, soxS, marR, acrA, QnrB6, CMY-46.	traT, irp2, fyuA	-
CP_Nsk3	AMC-CD-CPD-FOX	msbA, kdpE, PmrF, baeR, mdtC, mdtB, mdfA, marA, rsmA, KpnE, KpnF, CRP, ampH, emrB, emrR, H-NS, mdtG, UhpT, PBP3, GlpT, EF-Tu, soxS, marR, acrA, QnrB18, CMY-77, CMY-108.	-	-
CP_Nsk4	AMP-AMC-CPD-FOX	msbA, kdpE, PmrF, baeR, mdtC, mdtB, mdfA, marA, rsmA, KpnE, KpnF, CRP, ampH, emrB, emrR, H-NS, mdtG, UhpT, PBP3, GlpT, EF-Tu, soxS, marR, acrA, acrB, QnrB57, CMY-124.	-	-
CP_Nsk5	AK	PmrF, mdfA, acrA, rsmA, KpnE, KpnF, KdpE, mdtB, mdtC, baeR, msbA, CRP, marA, H-NS, CMY-46, ampH, emrR, emrB, QnrB17, mdtG, GlpT, UhpT, PBP3, EF-Tu, soxS, marR.	-	-
CP_Nsk6	AMP-AMC-CPD-FOX	KdpE, PmrF, mdfA, ampH, acrB, acrA, CRP, QnrB18, H-NS, mdtC, baeR, marA, msbA, mdtG, KpnE, KpnF, CMY-34, rsmA, emRB, emrR, GlpT, PBP3, UhpT, EF-Tu, soxS, marR, qnrB23, qnrB29, qnrB48, blaCMY-106, blaCMY-108, blaCMY-37	-	-
CP_Nsk7	AMC-CN-CPD-FOX-CTX	CMY-63, KdpE, QnrB6, acrA, PmrF, KpnF, KpnE, emrB, emrR, rsmA, msbA, CRP, baeR, mdtC, mdtB, H-NS, mdtG, mdfA, marA, ampH, UhpT, PBP3, GlpT, EF-Tu, soxS, marR, qnrB41, blaCMY-46	-	-
CP_Igbk1	AMP-AMC-CD-CPD-FOX	msbA, kdpE, PmrF, baeR, mdtC, mdtB, mdfA, marA, rsmA, KpnE, KpnF, CRP, ampH, emrB, emrR, H-NS, UhpT, PBP3, GlpT, EF-Tu, soxS, marR, acrA, QnrB6, qnrB9, blaCMY-25, blaCMY-124, blaCMY-2	-	-
CP_Igbk2	AMP-AMC-FOX	msbA, kdpE, PmrF, baeR, mdtC, mdtB, mdfA, marA, rsmA, KpnE, KpnF, CRP, ampH, emrB, emrR, H-NS, mdtG, UhpT, PBP3, GlpT, EF-Tu, soxS, marR, acrA, QnrB17, CMY-129,	-	-
CP_Igbk3	AMP-AMC-CD-CPD-FOX	msbA, kdpE, PmrF, baeR, mdtC, mdtB, mdfA, marA, rsmA, KpnE, KpnF, CRP, ampH, emrB, emrR, H-NS, mdtG, UhpT, PBP3, GlpT, EF-Tu, soxS, marR, acrA, QnrB6, CMY-35, qnrB9, blaCMY-2	-	-
CP_Igbk4	AMP-AMC-CD-CPD-FOX	msbA, kdpE, PmrF, baeR, mdtC, mdtB, mdfA, marA, rsmA, KpnE, KpnF, CRP, ampH, emrB, emrR, H-NS, mdtG, UhpT, PBP3, GlpT, EF-Tu, soxS, marR, acrA, QnrB18, CMY-124, qnrB48, qnrB23, qnrB29	-	-
CP_Igbk5	AMP-CPD-FOX	msbA, kdpE, PmrF, baeR, mdtC, mdtB, mdfA, marA, rsmA, KpnE, KpnF, CRP, ampH, emrB, emrR, H-NS, mdtG, UhpT, PBP3, GlpT, EF-Tu, soxS, marR, acrA, QnrB6, CMY-71	-	-
CP_Igbk6	AMP-AMC-CPD-FOX	msbA, kdpE, baeR, mdtC, mdtB, mdfA, marA, rsmA, KpnE, KpnF, CRP, ampH, emrB, emrR, H-NS, mdtG, UhpT, PBP3, GlpT, EF-Tu, soxS, marR, acrA, QnrB16, CMY-106	-	-
CP_Igbk7	FOX	msbA, kdpE, PmrF, baeR, mdtC, mdtB, mdfA, rsmA, KpnE, KpnF, CRP, ampH, emrB, emrR, H-NS, mdtG, UhpT, PBP3, GlpT, EF-Tu, soxS, marR, acrA, QnrB16, CMY-124	irp2, fyuA	-
CP_Igbk8	AMP-FOX	msbA, kdpE, PmrF, baeR, mdtC, mdtB, mdfA, marA, rsmA, KpnE, KpnF, CRP, ampH, emrB, emrR, H-NS, mdtG, UhpT, PBP3, GlpT, EF-Tu, soxS, marR, acrA, QnrB16, CMY-106	-	-
CP_Igbk9	-	msbA, kdpE, PmrF, baeR, mdtC, mdtB, mdfA, marA, rsmA, KpnE, KpnF, CRP, ampH, emrB, emrR, H-NS, mdtG, UhpT, PBP3, GlpT, EF-Tu, soxS, marR, acrA, QnrB17, CMY-77	-	-
CP_Igbk10	-	msbA, kdpE, PmrF, baeR, mdtC, mdtB, mdfA, marA, rsmA, KpnE, KpnF, CRP, ampH, emrB, emrR, H-NS, mdtG, UhpT, PBP3, GlpT, EF-Tu, soxS, marR, acrA, QnrB6, CMY-46	traT, irp2, fyuA	Col440I, IncFIB(K)
CP_Igbk11	AMC-FOX	msbA, kdpE, PmrF, baeR, mdtC, mdtB, mdfA, marA, rsmA, KpnE, KpnF, CRP, ampH, emrB, emrR, H-NS, mdtG, UhpT, PBP3, GlpT, EF-Tu, soxS, marR, acrA, QnrB18, CMY-108, qnrB48, qnrB23, qnrB29	-	-
CP_Igbk12	FOX	msbA, kdpE, PmrF, baeR, mdtC, mdtB, mdfA, marA, rsmA, KpnE, KpnF, CRP, ampH, emrB, emrR, H-NS, mdtG, UhpT, PBP3, GlpT, EF-Tu, soxS, marR, acrA, QnrB17, CMY-46, qnrB77	traT	IncFIB(pHCM2)
CP_Igbk13	AMC-CTX	KdpE, acrA, H-NS, mdtB, mdtC, baeR, rsmA, mdfA, CRP, emrB, emrR, PmrF, msbA, marA, KpnE, KpnF, QnrB6, ampH, mdtG, PBP3, UhpT, GlpT, EF-Tu, soxS, marR, qnrB13, blaCMY-46	irp2, fyuA, traT	-

AMP: ampicillin; AMC: amoxycillin/clavulanic acid; CN: gentamicin; AK: amikacin; CD: cefpodoxime/clavulanic acid; CPD: cefpodoxime; FOX: cefoxitin; CTX: cefotaxime.

## Data Availability

This Whole-Genome Shotgun project has been deposited at the DDBJ/ENA/GenBank under the BioProject accession no. PRJNA855947. The version described in this paper is the first version.
